# Utility of linking primary care electronic medical records with Canadian census data to study the determinants of chronic disease: an example based on socioeconomic status and obesity

**DOI:** 10.1186/s12911-016-0272-9

**Published:** 2016-03-11

**Authors:** Suzanne Biro, Tyler Williamson, Jannet Ann Leggett, David Barber, Rachael Morkem, Kieran Moore, Paul Belanger, Brian Mosley, Ian Janssen

**Affiliations:** Kingston, Frontenac, and Lennox & Addington Public Health, 221 Portsmouth Avenue, Kingston, ON K7M 1V5 Canada; Department of Community Health Sciences, University of Calgary, Calgary, AB Canada; The College of Family Physicians of Canada, Mississauga, ON Canada; Department of Family Medicine, Queen’s University, Kingston, ON Canada; Department of Public Health Sciences, Queen’s University, Kingston, ON Canada; School of Kinesiology and Health Studies, Queen’s University, Kingston, ON Canada; Department of Geography, Queen’s University, Kingston, ON Canada

**Keywords:** Socio-economic factors, Population health, BMI-Body Mass Index, EMR-electronic medical record, Obesity, Public health

## Abstract

**Background:**

Electronic medical records (EMRs) used in primary care contain a breadth of data that can be used in public health research. Patient data from EMRs could be linked with other data sources, such as a postal code linkage with Census data, to obtain additional information on environmental determinants of health. While promising, successful linkages between primary care EMRs with geographic measures is limited due to ethics review board concerns. This study tested the feasibility of extracting full postal code from primary care EMRs and linking this with area-level measures of the environment to demonstrate how such a linkage could be used to examine the determinants of disease. The association between obesity and area-level deprivation was used as an example to illustrate inequalities of obesity in adults.

**Methods:**

The analysis included EMRs of 7153 patients aged 20 years and older who visited a single, primary care site in 2011. Extracted patient information included demographics (date of birth, sex, postal code) and weight status (height, weight). Information extraction and management procedures were designed to mitigate the risk of individual re-identification when extracting full postal code from source EMRs. Based on patients’ postal codes, area-based deprivation indexes were created using the smallest area unit used in Canadian censuses. Descriptive statistics and socioeconomic disparity summary measures of linked census and adult patients were calculated.

**Results:**

The data extraction of full postal code met technological requirements for rendering health information extracted from local EMRs into anonymized data. The prevalence of obesity was 31.6 %. There was variation of obesity between deprivation quintiles; adults in the most deprived areas were 35 % more likely to be obese compared with adults in the least deprived areas (Chi-Square = 20.24(1), *p* < 0.0001). Maps depicting spatial representation of regional deprivation and obesity were created to highlight high risk areas.

**Conclusions:**

An area based socio-economic measure was linked with EMR-derived objective measures of height and weight to show a positive association between area-level deprivation and obesity. The linked dataset demonstrates a promising model for assessing health disparities and ecological factors associated with the development of chronic diseases with far reaching implications for informing public health and primary health care interventions and services.

## Background

Primary care practices have increasingly adopted electronic medical records (EMRs) to support clinical practice [1]. EMRs contain a breadth of longitudinal data including patient demographics, visit types, diagnosis codes for health conditions, physical measures, medications, diagnostic procedures, laboratory tests, referrals, immunizations, and risk factors [2, 3]. Researchers have recognised the potential for extracting EMR data to inform population health assessment, clinical research, data quality improvement initiatives and public health surveillance [2, 4–7]. One such repository is the Canadian Primary Care Sentinel Surveillance Network (CPCSSN).

Although CPCSSN was primarily designed to monitor chronic disease prevalence across Canada, it also provides an opportunity to examine the determinants of disease in an efficient manner. Research on the determinants of chronic disease typically involve the assembly of large cross-sectional samples and prospective cohorts [8]. The significant costs and participant burden associated with such studies, particularly studies with objective measures and/or large samples [8, 9], could be avoided by using a data source like CPCSSN.

Patient data from EMRs can be linked with other data sources, such as a postal code linkage with Census data, to obtain additional information on environmental determinants of health [10–12]. While promising, successful linkages between primary care EMRs with geographic measures as an approach for researching the determinants of chronic diseases is limited [13]. This in part reflects researcher and ethics review board concerns that extracting the geographic information from EMRs, such as full postal codes, that is required for linkages with electronic geographic information system (GIS) increases the risk of individual patient re-identification [14, 15].

This study tested the feasibility of enhancing existing CPCSSN primary care EMR data extraction algorithms to include full postal code, and to link this extracted data with area-level measures of the environment to demonstrate how such a linkage could be used to examine the determinants of disease. The aim of our study was to demonstrate the practicability and utility of linking across different databases to enhance the study of associations related to chronic diseases, and associated risk factors, with ecological factors known to enhance the promotion of health and the prevention of disease.

Our example is based on obesity, as obtained in the EMRs, and deprivation, as obtained in the area-level database linkage with the Census. We chose to use obesity in our example because it is a highly prevalent condition that is a major risk factor for several chronic diseases [16–19], and because there is existing evidence linking area-level socioeconomic status with obesity [20, 21]. Although we have examined the association between obesity and area-level deprivation in our example, the issues and approach we discuss are relevant to other determinants of disease and health outcomes.

## Methods

### Data sources

The CPCSSN offered a unique opportunity to address our objective because it is Canada’s first multi-disease EMR-based surveillance system [2]. CPCSSN standardizes primary care data extracted from multiple EMR platforms, from ten primary care practice-based research networks across the country. However, this feasibility study was limited to a single, primary care site. This allowed us to test REB approval of additional postal code data extraction, and to demonstrate whether a linked data set mitigated the risk associated with patient re-identification, or increased the risk of re-identification.

### Ethics approval and addressing privacy concerns

Approval for the study and confidentiality of patient data was obtained from the Queen’s University Health Sciences Research Ethics Board. Physicians provided written informed consent for a one-time extraction of patient full postal code and full date of birth. This data was added to the regularly extracted CPCSSN data (the CPCSSN data repository operates under pre-existing cross-jurisdictional REB approval processes) [22]. Working with the CPCSSN Research Privacy and Ethics Officer and the data manager at Kingston’s Practice Based Research Network of CPCSSN, algorithms were designed to determine if and how data extraction of full postal code from the OSCAR EMR vendor system and clinics could be done to meet the definition of “anonymized data”, as set out in the Tri-Council Policy Statement for the Ethical Conduct of Research Involving Humans (TCPS2) [23]. The TCPS2 is the federally required guideline used by research ethics boards across Canada to evaluate prospective research and the protection of research subjects from potential research-related harms, such as breach of privacy [23]. Information extraction and management procedures were designed to ensure that prior to entering into the CPCSSN’s central data repository, direct identifiers (name, health card number, for example) were not intentionally extracted but if found inadvertently in free text fields of the EMR, such information would be irrevocably stripped. No code or key that could re-identify the patient was stored with the CPCSSN researchers. A key was needed for stripping directly identifying patient information; however, the key was only made available and stored with the patient’s physician. Further steps were taken using algorithms to locate and remove other potential identifying information (physician name, for example) so the risk of re-identification from the remaining indirect identifiers (postal code, for example) would be low to very low.

CPCSSN employed third party de-identification software, PARAT [24]. Where a potential research query generated five or more data points, the software automatically removed one or more digits from a patient’s postal code, or changed the data of birth to an age range, until the research result was higher than five data points [25].

### Study sample

Our research sample included active adult patients, 20 years and older, of physicians from a primary health care physician group, between January 1^st^ and December 31^st^, 2011. The primary health care group is a comprehensive-health-team-based practice, 1 of 10 participating in Kingston Ontario’s Practice Based Research Network of CPCSSN. The practice is located in an urban centre (population ~ 150 000) serving patients from both urban and rural surrounding regions. Prior assessment revealed the population served in the practice has a proportionately higher number proportion of vulnerable patients with high material and social deprivation patients compared with by comparison to surrounding practices. Twenty-two physicians in the group practice use a common EMR, OSCAR, which contains all clinical and demographic data for each patient.

### Research data

Data extracted for this project also included patient sex, height and weight measurements, as well as observation date. The dataset excluded all cases with missing information, duplicate information, as well as height and weight measurements associated with pregnancy (measurements taken 9 months before and 12 months after the estimated date of birth). The dataset of patients with a BMI record was compared with excluded patients with missing BMI information using the eight CPCSSN chronic disease case definitions and age to determine whether there were significant differences between the dataset under study from the original extracted dataset.

Body mass index (BMI) was calculated as weight in kilograms (kg) divided by height in metres squared (m^2^). BMI was categorized using the adult BMI cut-points recognized by the World Health Organization as: underweight (<18.5 kg/m^2^), normal weight (18.5–24.9 kg/m^2^), overweight (25–29.9 kg/m^2^), and obese (≥30 kg/m^2^) [26]. Where an available weight measurement was documented without a corresponding height, the last height measurement per patient was used to calculate BMI. For patients with more than one BMI measure in 2011, the last measure was used. BMI measures <15 kg/m^2^ and >50 kg/m^2^ were excluded as outliers.

Area-based socio-economic (ABSE) measures were based on the Institute National de Santé Publique de Québec (INSPQ) index of material and social deprivation and the Canada 2006 Census of Population, and were derived using postal code data and the Statistics Canada Postal Code Conversion File. The combined deprivation index is a measure of socioeconomic status (SES) combining several ‘material’ and ‘social’ variables from the Canadian census (such as income, education, living alone or with a spouse, etc.) to derive a single measure of SES. The last year that the deprivation index was calculated was 2006 as the voluntary Canadian Household Survey in 2011 did not provide sufficient data to accurately calculate the deprivation index. To account for coverage of the practice patient population, the deprivation index was scaled to the Kingston, Frontenac and Lennox & Addington (KFL&A) Public Health Unit geographical boundaries. Deprivation index scores were assigned to quintiles where one (1) represented the least deprived and five (5) the most deprived for three components: combined material and social, material and social deprivation. The material component group indicators of education, employment and income, while the social component groups indicators related to marital status and family structure.

### Statistical analysis

All analyses were conducted using SAS software, version 9.3. To assess differences between the records with missing data and without missing data, the distribution of covariates among those with a BMI record and those without a BMI record were compared using chi-square tests for binary variables (eight chronic diseases in the CPCSSN database) and a t-test for age. The prevalence of different BMI categories were determined and expressed as proportions. Chi-square tests were used to determine the differences in obesity prevalence among deprivation quintiles. This test had 12° of freedom (4 for the deprivation quintiles x 3 for the BMI categories) and was considered significant at the 0.05 level. Absolute and relative differences between quintiles of deprivation and obesity were also calculated. The relationships between obesity and combined material and social deprivation were also determined after stratifying the sample by urban-rural status and into four age categories (20–39. 40–59. 60–79 and 80+ years). Chi-square tests were used to compare the proportions after stratifying the sample by urban-rural status and age categories.

## Results

### Privacy mitigation

All 22 physicians within the study primary health care group provided written informed consent for the one-time extraction of patient full postal code and full date of birth. This data was used in conjunction with extracted CPCSSN anonymized data from those physicians’ practices. The application of de-identification processes, along with the deployment of PARAT software ensured no additional risk of re-identification arose for patients.

### Sample characteristics

The dataset consisted of 30 147 observations from records of adult patients between January and December 2011, 7186 of whom were identified as unique patients. The data cleaning process excluded patients who were pregnant (*n* = 262, 4 % of total), missing heights or weights (*n* = 977, 14 %), had BMI measurements outside the 15–50 kg/^m2^ range (*n* = 63, 0.9 %), and had a within patient BMI variation for multiple visits of greater than 2 standard deviations (*n* = 3). Eighty-one percent of all patients had a valid BMI. Of those, a number of patients had postal codes that were outside of the KFL&A Public Health regional boundaries, erroneous or missing (*n* = 519, 9 %) and a further 298 patients had a postal code that did not match to a deprivation index score (5 %). The final study sample, comprised of 5022 unique patients with a valid BMI and assigned a deprivation index score, represented 70 % of the original dataset.

There were differences between the dataset under study and the original extracted dataset. Results indicate that those with BMI data were more likely to be male (59.3 % vs. 62.2 %, *p* = 0.0239), were older (53 vs. 47.5 years, *p* < .0001) and had higher rates of diabetes (13.7 % vs. 9.9 %, *p* < .0001), hypertension (27.4 % vs. 21.5 %, *p* < .0001), osteoarthritis (12.3 % vs. 8.5 %, *p* < .0001), COPD (17.9 % vs. 19.7 %, *p* = 0.0474), and epilepsy (1.9 % vs. 1.0 %, *p* = 0.0076) by comparison to those with missing BMI records.

Descriptive characteristics are in Table 1. Sixty percent were female. There were more patients in the most deprived quintile than the least deprived quintile. There were more patients who were socially deprived than materially deprived. Over two-thirds (64.3 %) had overweight or obesity. The association between obesity and combined material and social deprivation differed across age groups. There was a significant trend for only one age group, 40 to 59 years: the proportion of people with obesity increased with increasing deprivation. The trend appears to hold for both the 20–39 and 60–79 year age groups, but remains above the significant level threshold. For patients over 80 years, the power to detect a significant difference across patients was insufficient and remains inconclusive (Table 2). The association between obesity and combined material and social deprivation was different in urban versus rural patients. The prevalence of obesity increased with increasing level of deprivation for patients living in urban areas, while the power to detect a significant difference for patients living in rural areas was too weak (Table 3).

**Table 1:** Distribution of the 2011 sample by study variables

Category, *n* (%)	Total	Men	Women
	(*n* = 5022)	(*n* = 2043)	(*n* = 2979)
Age			
20–29 y	525 (10.5)	181 (9.0)	344 (11.5)
30–39 y	717 (14.3)	262 (12.7)	455 (15.3)
40–49 y	962 (19.2)	388 (19.0)	574 (19.0)
50–59 y	1023 (20.4)	440 (21.4)	583 (20.1)
60–69 y	901 (18.0)	406 (20.0)	495 (16.4)
70–79 y	590 (11.7)	251 (12.3)	339 (11.2)
≥ 80 y	304 (6.0)	115 (5.6)	189 (6.3)
Material deprivation score^a^			
1 (lowest deprivation)	1396 (27.8)	561 (27.5)	835 (28.0)
2	1238 (24.6)	502 (24.6)	736 (24.7)
3	820 (16.3)	360 (17.6)	460 (15.4)
4	796 (15.8)	342 (16.7)	454 (15.2)
5 (highest deprivation)	772 (15.4)	278 (13.6)	494 (16.6)
Social deprivation score^a^			
1 (lowest deprivation)	364 (7.2)	139 (6.8)	225 (7.5)
2	832 (16.6)	348 (17.0)	484 (16.2)
3	961 (19.1)	405 (19.8)	556 (18.7)
4	1026 (20.4)	417 (20.4)	609 (20.4)
5 (highest deprivation)	1839 (36.6)	734 (35.9)	1105 (37.1)
Body mass index			
Underweight	91 (1.8)	17 (0.8)	74 (2.5)
Normal weight	1700 (33.8)	527 (25.8)	1173 (39.4)
Overweight	1642 (32.7)	811 (39.7)	831 (27.9)
Obese	1589 (31.6)	688 (33.7)	901 (30.2)

^a^expected 20 % per quintile

**Table 2:** The relationship between obesity and combined material and social deprivation stratified by age group

Obese, *n* (%), *N* = 1589						
	Combined material and social deprivation quintile					
Age group, years, n	1 (least deprived)	2	3	4	5 (most deprived)	*p*-value
20–39 325	29 (8.9)	60 (18.5)	73 (22.5)	28 (8.6)	135 (41.5)	0.0863
40–59 713	99 (13.9)	173 (24.3)	130 (18.2)	69 (9.7)	242 (33.9)	<0.0001
60–79 499	61 (12.2)	134 (26.9)	137 (27.5)	35 (7.0)	132 (26.5)	0.0239
80+ 52	6 (11.5)	14 (26.9)	17 (32.7)	3 (5.8)	12 (23.1)	0.6081*

*small numbers decrease power and the ability to detect significant differences for patients in the oldest age group

**Table 3:** The relationship between obesity and combined material and social deprivation stratified by urban-rural status

Obese, *n* (%), *N* = 1589						
	Combined material and social deprivation quintile					
Region, *n*	1 (least deprived)	2	3	4	5 (most deprived)	*p*-value
Urban, 1324	180 (13.6)	232 (17.5)	277 (20.9)	120 (9.1)	515 (38.9)	<0.0001
Rural, 265	15 (5.7)	149 (56.2)	80 (30.2)	15 (5.7)	6 (2.3)	0.3904*

*small numbers decrease power and the ability to detect significant differences for rural patients

Assessing the relationship between obesity and the combined material and social deprivation, patients in the most deprived group were 35 % more likely to have obesity compared with patients in the least deprived group (Chi-Square = 20.24(1), *p* < 0.0001); this represented an absolute difference of 9.8 %. Table 4 shows different associations when the deprivation index is split into the two components of material and social deprivation. There were no differences in obesity across social deprivation quintiles, but there were significant differences across material deprivation quintiles. The most materially deprived group was 59 % more likely to have obesity compared with the least materially deprived group.

**Table 4:** Absolute and relative measures of disparity between the obesity prevelance for adults in the least and most deprived groups

		Absolute	Relative
Material deprivation level	Obesity prevalence (%)	Simple difference	Disparity rate ratio
1 (least deprived)	27.9	^a^	^a^
2	36.6	8.7	1.31
3	38.0	10.1	1.36
4	39.6	11.7	1.42
5 (most deprived)	44.4	16.5	1.59
Social deprivation level			
1 (least deprived)	37.6	^a^	^a^
2	36.2	−1.4	0.96
3	37.5	0	1.00
4	31.5	−6.1	0.84
5 (most deprived)	37.6	0	1.00

^a^The best group rate (least deprived) was used as the reference point

**Fig. 1:**
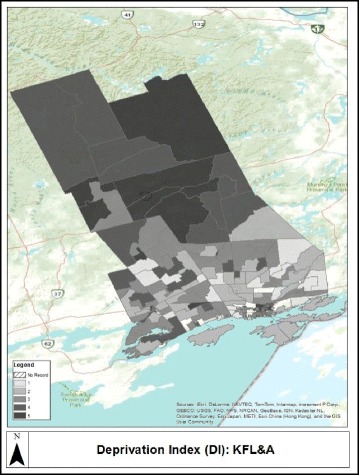
Relative deprivation status. The KFL&A Public Health regional boundaries as measured using the INSPQ Deprivation Index by dissemination areas using the 2006 census

**Fig. 2:**
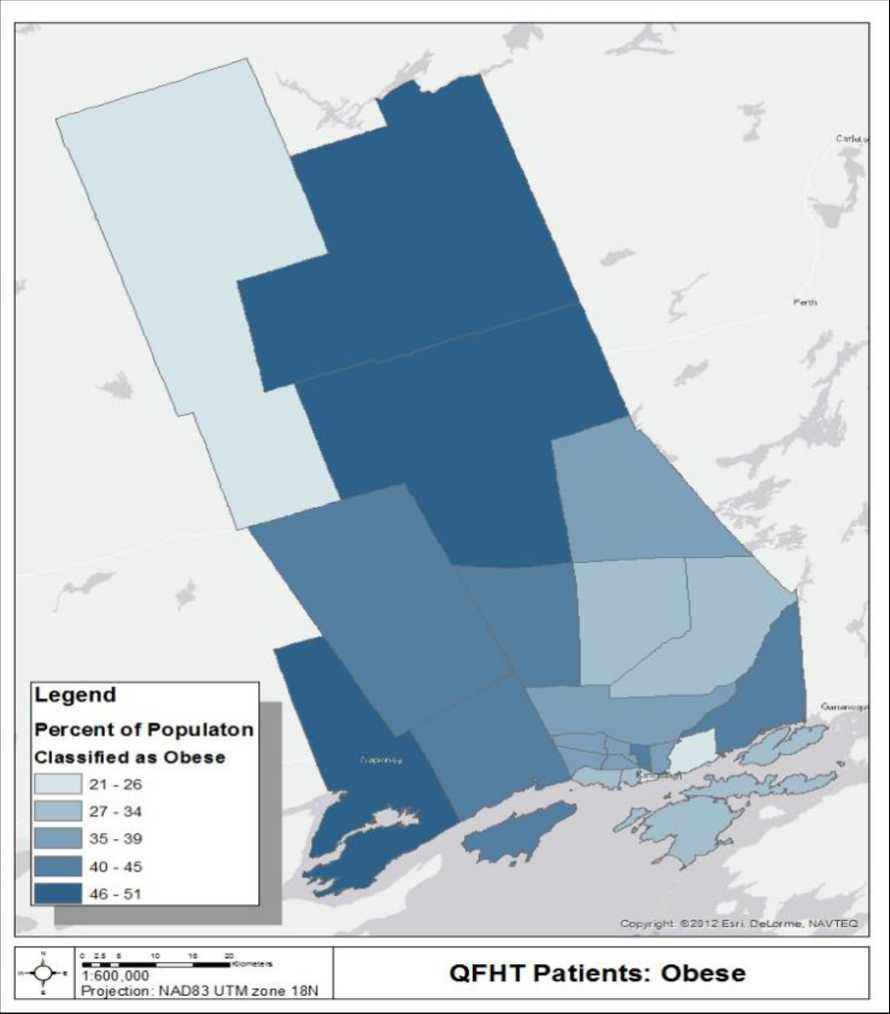
2011 patient study population classified as obese within the KFL&A Public Health regional boundaries

Figure 1 shows differences in the deprivation status for the KFL&A Public Health regional boundaries as measured using the 2006 census and Fig. 2 shows the spatial extent of the study population classified as obese within the KFL&A Public Health regional boundaries. Darker regions on both maps depict areas with higher deprivation and obesity prevalences, respectively.

## Discussion

This study demonstrates how primary care EMRs can be linked with census-based area-level measures of deprivation to examine the determinants of disease. To our knowledge, our team is the first in Canada to develop and implement linkage methods between a primary care chronic disease surveillance database with the Canada Census of Population [13]. With the addition of full postal code to a chronic disease surveillance database such as CPCSSN, there is an opportunity to assess chronic disease risk and protective factors in relation to socio-environmental neighbourhood contexts (e.g., aspects of the built environment that support increased active transportation; spatial associations between social service locations and areas of high rates of depression). Behavioural risk factors at the individual level (e.g., tobacco use, poor diet, physical inactivity, and excessive alcohol consumption) have a profound influence on the development and progression of chronic disease. Social determinants of health (e.g., occupation, ethnicity, level of education) affect health disparities. Yet currently socio-behavioural information is rarely captured, collected and used in an integrated, standardized way in primary care EMRs. It is our hope that primary care chronic disease surveillance will begin to incorporate these important determinant factors. As we move towards that enhancement, this study presents a methodology that promises to support database research that plays a vital role in identifying and understanding the complex factors tied to disparities in chronic disease prevalence and could inform place-based public health and primary health care intervention strategies anchored in prevention research [11, 27, 28]. The additional data extraction of full postal code and date of birth met TCPS2 ethical and technological requirements for rendering health information extracted from local EMRs into anonymized data. CPCSSN can manage the level of geographic suppression or aggregation in proportion to the risks of sharing particular datasets for the purposes of research and evaluation. To that end, the CPCSSN has unprecedented cross-jurisdictional and cross-provincial experience working with a variety of institutional research ethics boards and the variable provincial health information privacy legislation across Canada [22].

Integrating *Privacy by Design* principles into the design and architecture of a research project’s or organization’s privacy and information system protocols is the place to start for researchers, physicians and institutions. Evaluating risk findings as they arise against a protocol that reflects the organization’s tolerance for such risk serves as an early warning system to identify high-risk activities and mitigate the sources of such risk before an unwanted event arises. Following this research study, CPCSSN conducted a national, cross-jurisdictional overarching Privacy Impact Assessment and adopted *Privacy By Design* principles to include full postal code in EMR data extractions. REB-approved researchers can apply through CPCSSN’s data request protocol to conduct studies with the types of linkages and analyses presented in this paper.

Health inequalities are large in Canadian society and it is widely acknowledged that the environmental conditions in which we live are key determinants of our health [29]. Because 3 out of every 5 adult Canadians have a chronic disease and 4 out of every 5 are at risk of developing a chronic condition, there is an urgent need for chronic disease and associated risk factor research to account for the broader determinants of health when generating research investigations [30]. As an example, this study showed significant positive associations between deprivation and obesity. The association was attributable to material components of deprivation rather than the social components. This finding is consistent with earlier research [21, 31, 32]. This may reflect that the built environments in deprived neighbourhoods do not support healthy eating and physical activity to the same extent as the built environments in richer neighbourhoods [10, 33, 34]. Further, our results showing discrepancy between material and social deprivation point to the necessity of examining differing socioeconomic indicators, in context, to gain a better understanding of the patterns of association and their influence on risk for developing obesity [21].

As with any study, there are limitations that should be addressed. First, when using primary care EMRs for research it should be recognized that data were collected during patient/provider encounters using a system designed for patient care and not for research. Second, the study sample was comprised of individuals who visited their primary health care provider. This would influence the generalizability of our findings if the association between deprivation and obesity differs between individuals who do and do not visit a primary care physician. Third, because our aim was to test the feasibility of enhancing data extraction algorithms and to test the feasibility of linking EMRs to geographic measures, we did not control for potential confounders when examining the relationship between area-level deprivation and obesity. The relationship between obesity and deprivation in our study sample differed across age groups and between patients living in rural versus urban settings, illuminating the need for future research to consider additional underlying factors that are influencing health outcomes. Fourth, EMR data is plagued by missing and non-standardized data. Our study sample of patients with BMI records was slightly different compared with patients from the source data in terms of rates of chronic disease, sex and age. This would have introduced a selection bias in the present study if the association between area-level deprivation and obesity differed by these characteristics. Large variation in data quality has been shown to be more often attributable to practice based factors [35]. Though we designed data cleaning processes to mitigate erroneous data entry, it is possible that postal codes may have been entered with variations within each database that we were unable to detect, such that when merging across data sources these differences could have affected the accuracy of the study. Similar work conducted in the future could incorporate sensitivity analyses to account for missing data and explore the underlying factors driving data variability within the database.

## Conclusions

This study demonstrated that linking the CPCSSN anonymized health data with Canadian Census geography enables expanding investigations of the risks and protective factors for chronic diseases while safeguarding the privacy and security of patients. The study is a promising model for assessing health disparities and ecological factors associated with the development of chronic diseases. For both public health and primary health care, the ability to explore these associations has far reaching implications; the electronic architecture will ground health promotion and disease prevention strategies in empirical health evidence to support collective efforts to reduce health inequalities.

### Ethics approval and consent to participate

Research ethics approval was obtained from the Queen’s University Health Sciences Research Ethics Board. The CPCSSN project applies an opt-out protocol to remove anonymized patient information from the database of primary care electronic medical records. Physicians provided written informed consent for extraction of their patient’s anonymized health record data.

### Consent for publication

Not applicable.

### Availability of data and materials

The Canadian Primary Care Sentinel Surveillance Network (CPCSSN) is Canada’s first multi-disease surveillance system based on primary care electronic medical record (EMR) data. The data comes from physicians participating in 10 practice based research networks across Canada, extracted from multiple EMR systems. The data is extracted quarterly, mapped to a common database structure then cleaned and coded. Case detection algorithms are run against the dataset to identify individuals with one or more of eight chronic conditions (diabetes, hypertension, osteoarthritis, depression, chronic obstructive lung disease, dementia, Parkinson’s disease and epilepsy). The data within the CPCSSN database can be used to serve a number of different purposes and provide timely answers to relevant questions. The CPCSSN is housed at Queen’s University in Kingston, Ontario, Canada. For researchers who would like to include CPCSSN data as part of a research study, submitting a Letter of Intent is the first stage in the process. The process to obtain CPCSSN data for a research study is described in the “Research Using CPCSSN Data” schematic, which can be downloaded from the CPCSSN website at www.cpcssn.ca.

## Abbreviations

BMI: body mass index
COPD: chronic obstructive pulmonary disorder
CPCSSN: Canadian Primary Care Sentinel Surveillance Network
EMR: electronic medical record
GIS: geographic information system
REB: research and ethics board
SES: socioeconomic status
TCPS2: Tri-Council Policy Statement for the Ethical Conduct of Research Involving Humans
